# Model-Based Financial Consequences of Electrical Stimulation Therapy for Pressure Injury Healing

**DOI:** 10.3390/healthcare14101269

**Published:** 2026-05-07

**Authors:** Yoshiyuki Yoshikawa, Koji Ikeda, Mikiko Uemura, Noriaki Maeshige

**Affiliations:** 1 Department of Rehabilitation, Faculty of Health Sciences, Naragakuen University, Nara 631-8524, Japan; koji-ikeda@naragakuen-u.jp; 2 Department of Rehabilitation Science, Graduate School of Health Sciences, Kobe University, Kobe 654-0142, Japan; uemura@tamateyama.ac.jp (M.U.); nmaeshige@pearl.kobe-u.ac.jp (N.M.); 3 Department of Rehabilitation, Faculty of Health Science, Kansai University of Welfare Sciences, Osaka 582-0026, Japan

**Keywords:** pressure ulcer, electrical stimulation, budget impact, time to heal, DESIGN-R, implementation cost, monetary value proxy

## Abstract

**Highlights:**

**What are the main findings?**

- A model-based cost–consequence analysis translating 14-day randomized crossover trial data estimated that electrical stimulation (ES) accelerated short-term pressure injury healing (Δ = 0.128 cm^2^/day; *p* = 0.008).
- Using DESIGN-R– and depth-stratified median time-to-heal imputation, ES was estimated to reduce time to heal by 126.9 days and yield a gross monetary value offset of JPY 507,723/case, with modeled net financial impact remaining positive under the base-case assumptions after implementation costs (JPY 459,929/case).

**What are the implications of the main findings?**

- Facility-level adoption decisions can be informed by combining short-term healing dynamics with severity-stratified time-to-heal estimates, explicitly accounting for devices, consumables, and staff time.
- The estimated benefits were larger in higher-severity deep injuries (DESIGN-R ≥19), suggesting that severity-based prioritization may maximize modeled budget-impact consequences when resources are constrained.

**Abstract:**

**Background:** Electrical stimulation (ES) is recommended as an adjunctive therapy for pressure injuries (PIs), but adoption requires implementation-relevant estimates of facility-level budget impact, including delivery costs. **Methods:** We conducted a model-based financial consequence analysis (provider perspective, Japan) using a 14-day randomized double-blind crossover trial (*n* = 12). Baseline time to heal under placebo (T_placebo) was imputed from published median healing times stratified by DESIGN-R category and PI depth. A ratio-based healing acceleration parameter (*r*; treated as a secondary translation parameter) was used to estimate time to heal under ES (T_ES = T_placebo/*r*) and days saved. Days saved were valued as a modeled gross offset using JPY 4000/day as a healing-related monetary value proxy; ES implementation costs comprised device allocation, consumables, and incremental staff time; and uncertainty was propagated via 10,000 patient-level bootstrap resamples. **Results:** The primary crossover effect was Δ = 0.128 cm^2^/day (ES minus placebo; *p* = 0.008), and *r* was 3.52 (95% uncertainty interval [UI] 1.53–13.67). Under the base-case modeled translation, mean T_placebo was 177.3 days and mean T_ES was 50.4 days, yielding 126.9 days saved (95% UI 63.5–164.9). The modeled gross offset (proxy-based) was JPY 507,723/case (95% UI 253,854–659,434). Mean implementation cost was JPY 47,794/case, giving a net financial impact (proxy-based) of JPY 459,929/case (95% UI 158,459–640,086). **Conclusions:** Under the base-case-modeled assumptions, ES may be cost offsetting when healing-related care is valued using a monetary value proxy. However, prospective studies directly measuring time to heal and resource use are warranted.

## 1. Introduction

Pressure injuries (PIs) are a prevalent type of chronic wound that disproportionately affects populations with impaired mobility and/or sensation, including older adults, individuals with chronic diseases, and persons with spinal cord injuries. Once developed, PIs often require a prolonged period to heal [1,2]. Delayed healing has substantial clinical consequences: it increases the risk of infection, prolongs pain and activity limitations, and can adversely affect quality of life [3]. From a health-system perspective, prolonged healing also leads to sustained and multifaceted resource use, including repeated wound care procedures, use of pressure-redistributing support surfaces, staff time for assessments and dressing changes, and nutritional interventions [4]. Therefore, PIs represent not only a clinical challenge but also a major resource-allocation issue in healthcare and long-term care systems [5]. Importantly, time to heal is a core endpoint that simultaneously reflects patient-centered outcomes and healthcare resource consumption; interventions that shorten time to heal may yield clinical benefits while also reducing medical and long-term-care expenditures [2,5].

Electrical stimulation therapy has long been investigated as a physical therapy modality to promote wound healing in PIs. Clinical practice guidelines, including the Japanese Society of Pressure Ulcers guideline (5th edition), recommend electrical stimulation as an intervention for accelerating healing, indicating that its clinical effectiveness is supported to some extent [6]. However, even when the evidence base supports clinical effectiveness, decision-making in routine care often hinges on a different question: “How large is the expected economic impact at the ward or facility level once implementation costs are considered?” Evidence of clinical effectiveness alone does not quantify the magnitude of resource savings, nor does it specify whether potential savings outweigh the practical costs of delivering electrical stimulation (devices, consumables, and staff time). Although economic considerations are frequently discussed as one of several factors influencing adoption, robust, facility-interpretable estimates of cost consequences associated with electrical stimulation for PIs in Japan remain limited [7,8,9,10]. Accordingly, an important remaining gap is to translate established clinical effects into an explicit estimate of resource use and cost implications [9,11].

A key methodological step in economic evaluation is the objective estimation of time to heal [12]. Time to heal is commonly analyzed as a time-to-event outcome using survival methods (e.g., Kaplan–Meier curves and Cox proportional hazards models), supporting its use as a primary endpoint in wound research [13]. DESIGN-R is a PI assessment scale developed in Japan and widely used to standardize clinical evaluation of wound severity and healing status [14]. Prior work has reported median time to heal stratified by DESIGN-R total score (and PI depth), providing a practical basis for assigning baseline time to heal by severity strata [15]. This offers a useful bridge for translating short-term trial outcomes (e.g., wound area reduction over a defined period) into longer-term outcomes (time to heal) and subsequently into cost consequences [16]. Nevertheless, because time to heal is rarely measured directly in short trials, transparent modeling choices—and explicit characterization of uncertainty—are essential for producing decision-oriented estimates.

Therefore, the present study aimed to estimate the potential cost impact of electrical stimulation therapy for PIs as an implementation-relevant evidence synthesis rather than as advocacy for a specific therapy. More specifically, we addressed the pragmatic question of how large the expected reduction in healing time and the corresponding modeled financial consequences might be when expressed in a form interpretable for routine care. To address this question, we first performed a secondary analysis of data from a randomized crossover trial evaluating the healing-promoting effect of electrical stimulation in PIs (Yoshikawa et al.) [17] using a crossover-appropriate within-subject contrast as the primary effect estimate. We then used a ratio-based healing acceleration parameter as a secondary translation parameter to map the short-term clinical effect onto estimated time to heal, and imputed baseline time to heal using published median values stratified by DESIGN-R category and PI depth (Sanada et al.) [15]. Finally, we translated the estimated reduction in healing time into modeled gross offsets by applying a per-day cost representing PI-related care (e.g., wound procedures, labor costs, pressure-redistributing support surfaces, and nutritional supplementation). Because these values were based on fee-schedule–anchored and pragmatic unit-cost assumptions, they were treated as provider-relevant economic proxies rather than as directly observed reimbursement or true accounting costs. We further incorporated direct implementation costs of electrical stimulation (device allocation, consumables, and incremental staff time) to estimate the modeled net financial impact and the break-even implementation cost. Rather than conducting a full cost-effectiveness analysis, this study adopted a per-case cost–consequence framework to quantify modeled healing-related cost offsets attributable to fewer healing days.

We hypothesized that electrical stimulation would accelerate wound area reduction compared with placebo, leading to shorter estimated time to heal and lower PI-related costs. Furthermore, because higher baseline DESIGN-R scores indicate greater severity and longer expected healing times [15], the same relative healing acceleration would be expected to translate into larger absolute reductions in healing days in more severe injuries. Therefore, we hypothesized that more severe PIs may yield larger absolute reductions in healing days and, consequently, greater modeled gross offsets, supporting the potential for severity-based prioritization. Given the uncertainty inherent in extrapolating short-term healing dynamics, we additionally expected that uncertainty intervals would be informative for interpreting the plausibility and range of economic consequences.

## 2. Materials and Methods

### 2.1. Study Design

This study was a model-based cost–consequence analysis designed as a facility- or ward-level, budget-impact-oriented translation of clinical evidence in Japan. The analytic perspective was the healthcare provider (ward/facility) financial perspective, focusing on the expected monetary consequences associated with shortening the healing period when implementing electrical stimulation (ES) for pressure injuries (PIs). Because direct micro-costing was not available, healing-related resource use per day was valued using a fee-schedule–anchored proxy combined with pragmatic unit-price assumptions, including wage-based labor costs, support-surface rental/list prices, and acquisition costs for nutritional supplementation. These values were, therefore, treated as provider-relevant economic proxies rather than as directly observed reimbursement amounts or true accounting costs. Accordingly, all monetary outcomes should be interpreted as facility-relevant financial proxies rather than true economic costs.

The time horizon was the estimated period until PI healing. All monetary values were expressed in Japanese yen (JPY) as nominal values (no inflation adjustment). We explicitly parameterized ES implementation costs (device allocation, consumables, and incremental staff time) and incorporated them into net impact and threshold (break-even) analyses. Because the clinical input was derived from a randomized crossover trial, the primary effectiveness estimation used a crossover-appropriate within-subject contrast, while ratio-based summaries were treated as secondary/descriptive measures for translation. Accordingly, net financial impact was interpreted as a modeled facility-level financial consequence under stated assumptions rather than as a directly observed cost saving.

### 2.2. Data Sources

Clinical effectiveness inputs were derived from a previously reported randomized crossover trial conducted by Yoshikawa et al., which enrolled 12 patients with PIs [17]. In that trial, both the placebo period and the ES period lasted 14 days. The present study was a secondary economic modeling analysis of those previously reported clinical data and addressed a distinct question: how short-term healing dynamics might translate into implementation-relevant healing-time consequences and facility-level financial implications. To assign baseline time to heal under placebo/usual care conditions, we used published median healing times stratified by DESIGN-R total score category and PI depth, as reported by Sanada et al. [15]. Because these values were external median estimates rather than directly observed mean healing times in the present trial population, they were treated as structural model inputs and were further examined in sensitivity analyses. The within-subject difference in daily wound area reduction (Δ) was regarded as the primary crossover-appropriate estimate of short-term clinical effectiveness. In contrast, the healing acceleration ratio (*r*) was not treated as a primary effect estimate, but only as a pragmatic translation parameter used to link short-term wound-healing dynamics to imputed time to heal in the economic model. Thus, Δ represented the primary short-term clinical effect, whereas *r* served only to translate that effect into longer-term modeled healing-time consequences.

### 2.3. Definitions of Main Variables and Outcomes

#### 2.3.1. Daily Wound Area Reduction

For each period, daily wound area reduction (cm^2^/day) was calculated as:

Daily wound area reduction = (wound area at start of period − wound area at end of period)/14

#### 2.3.2. Primary Effectiveness Estimate (Within-Subject Difference)

To reflect the crossover design, the primary effect size was defined as the within-subject difference in daily wound area reduction between periods:

Δ = (daily wound area reduction during electrical stimulation period) − (daily wound area reduction during placebo period).

We summarized Δ using the mean difference and its 95% confidence interval (CI). The null hypothesis, that mean Δ = 0, was evaluated using a paired t-test as the primary analysis and the Wilcoxon signed-rank test as a robustness check. As a crossover-design diagnostic, we additionally examined possible period and sequence effects using a simple crossover framework, recognizing the limited statistical power of such analyses in a 12-patient study. These diagnostic estimates were provided primarily for transparency rather than for definitive inference.

#### 2.3.3. Secondary Effectiveness Summary (Healing Acceleration Ratio)

The healing acceleration ratio was defined as:

*r* = (mean daily wound area reduction during ES)/(mean daily wound area reduction during placebo).

This ratio-based metric was treated as a secondary descriptive translation parameter rather than as the primary causal estimate because ratio measures may become unstable when the denominator is small or negative [18]. Accordingly, the primary short-term clinical effect was summarized by Δ, whereas *r* was retained only to provide a pragmatic link between short-term wound-area dynamics and the external time-to-heal framework used in the economic model.

Because the assumption of constant proportional acceleration throughout the full healing trajectory may be overly strong, especially in deep or clinically complex PIs, *r*-based translation was interpreted as a base-case approximation rather than as a biological representation of the full healing course. To examine the stability and conservativeness of this translation, we prespecified sensitivity analyses using nonparametric bootstrap resampling, median-based and trimmed-ratio scenarios, and a conservative diminishing-acceleration scenario.

#### 2.3.4. Baseline Time to Heal (Stratified Median Imputation)

Baseline time to heal under placebo/usual care was imputed using stratified published median healing times from Sanada et al. according to DESIGN-R total score category and PI depth [15]. DESIGN-R categories were defined as ≤9, 10–18, and ≥19 points. PI depth was categorized as superficial (d1–d2) or deep (D3–D5). The median healing times used were as follows:

- Superficial PIs: 15, 33, 140 days (for DESIGN-R ≤ 9, 10–18, ≥ 19)
- Deep PIs: 26, 63, 259 days (for DESIGN-R ≤ 9, 10–18, ≥ 19)

These published median values were used as imputed baseline healing times in the model rather than as directly observed trial outcomes.

#### 2.3.5. Counterfactual Estimated Time to Heal Under Electrical Stimulation

In the base-case analysis, estimated time to heal under ES was calculated as:

Estimated time to heal under ES = imputed baseline time to heal/*r*.

This formulation was used as a transparent base-case translation from short-term healing dynamics to longer-term healing-time consequences. However, because wound healing is not necessarily linear over the full course of recovery, particularly in deep and clinically complex PIs, this base-case translation should be interpreted as a simplifying modeling assumption rather than as a biological law.

To assess the dependence of the results on this assumption, we prespecified conservative alternative translation scenarios. First, we examined a diminishing-acceleration scenario, in which the proportional acceleration observed during the 14-day ES period was assumed to weaken during later healing. Second, we explored supplementary alternative summaries of short-term treatment benefit to assess whether the direction and approximate magnitude of projected time savings were preserved without relying solely on the base-case ratio formulation.

#### 2.3.6. Days Saved

Days saved were defined as:

Days saved = Imputed baseline time to heal − Estimated time to heal under stimulation.

#### 2.3.7. Gross Monetary Value Offset

A per-day healing-related monetary value (JPY/day) was specified and multiplied by days saved to estimate the modeled gross offset per case:

Gross offset (JPY/case) = days saved × per-day healing-related monetary value.

The per-day healing-related monetary value was constructed as a facility-relevant proxy for healing-related resource use, anchored partly to the Japanese fee schedule and partly to pragmatic unit-price assumptions. Importantly, fee schedule amounts reflect reimbursement/charges and do not necessarily equal true economic costs. Therefore, the resulting per-day value should be interpreted as a budget-impact-oriented proxy rather than a societal or accounting “true cost.”

The base-case per-day healing-related monetary value was set at JPY 4000/day, comprising the following components:

- Local wound procedures (JPY 1000/day): anchored to the reimbursement schedule for severe PI procedures (J001-4), converted using the standard fee-schedule conversion (points × JPY 10) and rounded pragmatically for modeling [19].
- Labor (JPY 1500/day): estimated using a time × wage approach, assuming 30 min/day of PI-related care and JPY 50/min [20].
- Support surfaces (JPY 500/day): estimated by converting a monthly rental/list cost of JPY 15,000/month to JPY 500/day.
- Nutritional supplementation (JPY 1000/day): estimated using assumed acquisition costs and dosing (JPY 500 per serving × 2/day) [21].

These components were intended to approximate healing-related resource inputs from the provider perspective. They should not be interpreted as direct measures of actual healthcare expenditure for every setting. Because this per-day value is a proxy and may not scale perfectly linearly with healing days, it was varied over a wider range in sensitivity analyses.

#### 2.3.8. ES Implementation Cost

To reflect real-world delivery, direct ES implementation costs were parameterized as device acquisition, per-patient consumables, and incremental staff time attributable specifically to ES delivery. The ES device cost was JPY 77,000. Because a single device can be used across multiple patients, we assumed that one device covered all 12 cases and allocated the device cost equally across cases, yielding JPY 6417 per case (JPY 77,000/12). This approach was selected as a transparent and conservative trial-anchored assumption that did not rely on uncertain facility-specific inputs such as annual patient volume or device useful life.

Consumables were assumed to be patient-specific and single-use per case: one lead wire/cable (JPY 1800) and one pack of electrodes (8 pieces; JPY 1800), totaling JPY 3600 per case. Additional staff time specifically attributable to ES delivery was assumed to be 10 min before treatment and 5 min after treatment (15 min/day in total). Using the same wage assumption of JPY 50/min, the incremental labor cost of ES delivery was JPY 750/day. For analyses requiring a per-day implementation cost during the modeled healing period under ES, fixed per-case items were amortized evenly across the estimated time to heal under ES, yielding:

C_impl/day = [(device allocation + consumables)/(estimated time to heal under ES)] + incremental staff time per day.

#### 2.3.9. Interpretation of Gross Offsets and Threshold Analysis

The gross offset represents the gross facility-relevant monetary value associated with fewer healing days and, by definition, does not subtract the direct implementation costs of ES. We therefore evaluated the net financial impact by incorporating ES implementation costs. Net financial impact per case was defined as:

Net financial impact (JPY/case) = (days saved × per-day healing-related monetary value) − (C_impl/day × T_ES),

where T_ES denotes the estimated time to heal under ES.

When implementation costs were expressed as fixed per-case and variable per-day components, the same quantity was equivalently written as:

Net financial impact (JPY/case) = (days saved × per-day healing-related monetary value) − [(device allocation + consumables) + (incremental staff time per day × T_ES)].

Because both healing-time translation and monetary valuation relied on model assumptions and proxy-based costing, net financial impact was interpreted as a modeled facility-level consequence under stated assumptions rather than as an observed economic saving. The break-even implementation cost per ES treatment day was defined as the maximum average implementation cost that would reduce net financial impact to zero under the model assumptions.

### 2.4. Sensitivity Analyses

Because model estimates depend on the per-day healing-related monetary value, the translation of short-term healing dynamics into time to heal, and the imputed baseline healing time derived from published severity-stratified medians, we prespecified the following sensitivity analyses.

#### 2.4.1. One-Way Sensitivity Analysis for Per-Day Monetary Values

The base-case per-day healing-related monetary value (JPY 4000/day) was varied across a wider plausible range of JPY 3000, 5000, 7000, 8000, 9000, and 10,000/day, and the resulting changes in gross offset and net financial impact were evaluated.

#### 2.4.2. Sensitivity Analyses for the Healing Acceleration Ratio

We examined the stability of the ratio-based translation parameter using three complementary approaches.

(a) Nonparametric bootstrap: A patient-level nonparametric bootstrap with replacement (10,000 iterations) was performed. In each iteration, *r* was recalculated as the ratio of the mean daily wound area reduction during ES to that during placebo, and all downstream model outputs were recomputed.
(b) Median-based and trimmed-ratio scenarios: To assess the influence of skewness and extreme values, we additionally examined a median-based ratio and a trimmed-ratio scenario. In the trimmed-ratio scenario, bootstrap-derived *r* values below the 2.5th percentile and above the 97.5th percentile of the bootstrap distribution were excluded. These analyses were intended as robustness checks rather than replacements for the primary base-case analysis.
(c) Deterministic variation around the base-case *r*: For interpretability, *r* was also varied by ±20% around the base-case point estimate, and the corresponding changes in estimated time to heal, days saved, gross offset, and net financial impact were reported.

#### 2.4.3. Conservative Alternative Translation Scenario

Because the base-case model assumes constant proportional acceleration across the full healing trajectory, we additionally evaluated a conservative diminishing-acceleration scenario in which the observed short-term acceleration was assumed to attenuate during later healing.

#### 2.4.4. Sensitivity Analysis for Imputed Baseline Time to Heal

Because baseline time to heal under placebo was assigned from published severity-stratified median values, we evaluated the dependence of the results on this external input by varying the imputed baseline healing time by ±20% and ±30%. The corresponding effects on estimated time to heal under ES, days saved, gross offset, and net financial impact were reported.

#### 2.4.5. Structural/Subgroup Sensitivity Based on DESIGN-R Strata

As the primary subgroup presentation, outputs were estimated according to DESIGN-R category and PI depth using the corresponding stratified baseline median healing times. As an additional structural sensitivity analysis, we examined an alternative scenario without depth stratification to evaluate the influence of depth handling on the model outputs.

## 3. Results

### 3.1. Crossover Diagnostics

In an exploratory 2 × 2 crossover model adjusting for period and sequence, the direction and magnitude of the treatment effect were consistent with the paired within-subject estimate (Δ). No material period or sequence effects were observed. Carryover assessment, based on a previous treatment term and/or treatment-by-period interaction, did not suggest a clinically meaningful carryover effect; however, these checks were underpowered because of the small sample size. A first-period-only sensitivity analysis yielded a treatment effect in the same direction. For transparency, treatment, period, and sequence effects are provided in Table S1.

### 3.2. Primary Crossover-Appropriate Effectiveness Estimate

Across the 12 cases, the mean within-subject difference in daily wound area reduction was Δ = 0.128 cm^2^/day (electrical stimulation minus placebo; 95% CI 0.041–0.216; paired *t*-test *p* = 0.008), indicating faster short-term wound area contraction during electrical stimulation. Because this within-subject contrast directly reflects the randomized crossover comparison and is less sensitive than ratio-based measures to negative or near-zero placebo-period changes, Δ was treated as the primary clinical effectiveness estimate. As a robustness check, Wilcoxon signed-rank test yielded a consistent result (*p* = 0.028).

### 3.3. Secondary Ratio-Based Summary for Economic Translation

To translate the short-term crossover effect into an implementation-relevant estimate of time to heal, we additionally summarized the data using the healing acceleration ratio (*r*) as a pragmatic translation parameter rather than as the primary causal estimate. Across the 12 cases, the mean daily wound area reduction was 0.179 cm^2^/day during the electrical stimulation period and 0.051 cm^2^/day during the placebo period, yielding a base-case healing acceleration ratio of 3.52. Using a patient-level nonparametric bootstrap resampling (10,000 iterations), the 95% uncertainty interval for *r* was 1.53 to 13.67, indicating substantial uncertainty and right skewness in the ratio-based translation.

Because the placebo period mean reduction was small, some bootstrap iterations produced relatively large *r* values. These values were not treated as errors and were not removed from the primary bootstrap analysis because they reflect genuine sampling instability inherent to ratio-based summaries in a small sample. Instead, their influence was explicitly examined through supplementary sensitivity analyses, including median-based and trimmed-ratio scenarios and a conservative diminishing-acceleration scenario.

These supplementary analyses included an alternative summary statistic (median-based ratio) and more conservative scenarios (trimmed-ratio and diminishing-acceleration scenarios). To further examine the stability of the ratio-based translation parameter, supplementary sensitivity analyses were conducted using a median-based ratio and a trimmed-ratio scenario (Table S2). The median-based ratio was 6.00, whereas the trimmed ratio was 4.24. When applied to the economic model, these alternative ratios yielded estimated times to heal under ES of 29.6 and 41.8 days, corresponding to 147.8 and 135.5 days saved, respectively. The corresponding modeled gross offsets were JPY 591,000 and JPY 542,093 per case, and the corresponding modeled net financial impacts were JPY 558,821 and JPY 500,744 per case. Although the absolute magnitudes of the projections varied across ratio summaries, the direction of the modeled effect remained unchanged. Similarly, in the conservative diminishing-acceleration scenario, estimated days saved and modeled monetary consequences were attenuated relative to the base case, while the overall interpretation remained directionally similar. Taken together, these findings support interpreting *r* as a pragmatic translation parameter for scenario-based modeling rather than as a precise or biologically complete representation of the full healing trajectory.

### 3.4. Base-Case Modeled Time to Heal and Monetary Consequences

Based on DESIGN-R total scores, the mean imputed baseline time to heal under placebo/usual care was 177.3 days. Applying the base-case healing acceleration ratio to the baseline predictions, the estimated time to heal under electrical stimulation was 50.4 days, corresponding to 126.9 days saved. The bootstrap-derived 95% uncertainty intervals were 12.4 to 113.8 days for estimated time to heal under stimulation and 63.5 to 164.9 days for days saved. Assuming the base-case per-day healing-related monetary value proxy of JPY 4000/day, the modeled gross offset was JPY 507,723 per case, with a bootstrap-derived 95% uncertainty interval of JPY 253,854 to 659,434. When electrical stimulation implementation costs were incorporated, including the device allocated across 12 cases, patient-specific consumables, and additional staff time, the estimated implementation cost was JPY 47,794 per case (including JPY 10,017 fixed per-case cost and JPY 37,777 variable labor cost based on 50.4 days × JPY 750/day). The resulting modeled net financial impact was JPY 459,929 per case (bootstrap-derived 95% uncertainty interval: JPY 158,459 to 640,086). Because both healing-time translation and monetary valuation relied on model assumptions and proxy-based costing, these results should be interpreted as modeled facility-level financial consequences under the stated assumptions rather than as directly observed economic savings. Under the base-case assumptions, however, the model suggested that electrical stimulation remained potentially cost offsetting after implementation costs were included. The corresponding point estimates and 95% uncertainty intervals are summarized in Table 1.

### 3.5. Sensitivity Analyses

Sensitivity analyses varying the imputed baseline time to heal demonstrated that the magnitude of the projected model outputs depended, as expected, on the externally sourced baseline healing-time input, whereas the direction of the results remained unchanged. When baseline time to heal was varied by ±20% and ±30%, estimated days saved, gross offset, and net financial impact changed proportionally, confirming that baseline healing-time assumptions materially influenced the absolute scale of the projected outcomes. These findings support interpreting the reported monetary estimates as scenario-based projections rather than fixed forecasts. Similarly, one-way sensitivity analyses varying the per-day healing-related monetary value proxy across JPY 3000, 5000, 7000, 8000, 9000, and 10,000/day showed that the modeled net financial impact depended on the assumed daily monetary value, but the direction of the modeled consequence remained unchanged across the examined range (Table S3). Together with the alternative ratio-based and conservative translation scenarios, these analyses indicate that the qualitative interpretation of the model was robust to a range of plausible assumptions, although the absolute magnitude of projected monetary consequences varied materially across scenarios.

### 3.6. Break-Even Implementation Cost

Using the base-case per-day healing-related monetary value proxy (JPY 4000/day) and the point estimate of the healing acceleration ratio (3.52), the break-even average implementation cost of electrical stimulation was JPY 10,080 per treatment day. In the base case, the estimated average implementation cost was approximately JPY 949 per treatment day (JPY 47,794/50.4 days). Thus, under the base-case model assumptions, the estimated implementation cost remained well below the break-even threshold (Table 2).

## 4. Discussion

This study used a model-based approach that combined a secondary analysis of randomized crossover trial data with published DESIGN-R and depth-stratified median time-to-heal estimates (stratified median imputation) to estimate the potential facility-level financial consequences of electrical stimulation (ES) therapy for pressure injuries (PIs) in Japan [15,17]. Consistent with the crossover design, the primary clinical effect was estimated using a within-subject difference in short-term wound area contraction (Δ). By contrast, the ratio-based healing acceleration parameter (*r*) was used as a secondary translation parameter to map short-term healing dynamics onto modeled time to heal within the economic analysis.

A key point for interpretation is that the monetary outputs in this study reflect a provider (ward/facility) financial perspective and were valued using a fee-schedule–anchored proxy combined with pragmatic unit-price assumptions rather than direct micro-costing. Therefore, the estimated “cost offsets” should be interpreted as facility-relevant budget-impact proxies attributable to fewer healing days (i.e., reduced intensity/duration of healing-related inputs), not as true economic costs at the societal level [19,20,21]. Using the 14-day wound area reduction rate, the healing acceleration ratio was 3.52, and the mean estimated time to heal decreased from 177.3 days at baseline to 50.4 days under electrical stimulation, corresponding to 126.9 days saved. Under the base-case assumption of JPY 4000 per day for healing-related care, the estimated gross offset (proxy-based) was approximately JPY 0.51 million per case (JPY 507,723). When direct implementation costs of electrical stimulation were incorporated (device cost allocated across 12 cases, patient-specific consumables, and additional delivery time), the mean implementation cost was JPY 47,794 per case (≈JPY 949 per treatment day). This yielded a modeled net financial impact of JPY 459,929 per case. Given that time to heal is a major driver of cumulative resource use in PI care, translating a clinically interpretable outcome (healing acceleration) into a decision-relevant metric (cost consequence) is a key contribution of this study [2,5,21,22,23]. Importantly, bootstrap-based uncertainty propagation suggested that the direction of the effect was consistently associated with positive modeled gross offsets in healing-related care, with the 95% uncertainty interval for the gross offset ranging from approximately JPY 0.25 to 0.66 million per case (JPY 253,854 to 659,434). After accounting for implementation costs, the modeled net financial impact remained positive within the base-case assumptions (95% uncertainty interval: JPY 158,459 to 640,086).

A notable feature of the present sample is that all injuries were classified as deep (D3–D5). Deep PIs generally require longer healing times and sustained, multidisciplinary management, including local wound care, pressure redistribution, and nutritional assessment/intervention [1,4,24]. Consequently, if a similar relative healing acceleration is achieved, cases with longer baseline healing times would be expected to yield larger absolute reductions in healing days and greater modeled gross offsets. Consistent with this rationale, severity-stratified estimates based on DESIGN-R indicated larger absolute benefits in higher-severity injuries: the DESIGN-R 10–18 group showed 45.1 days saved and an estimated gross offset of JPY 180,409 per case, whereas the DESIGN-R ≥ 19 group showed 185.4 days saved and an estimated gross offset of JPY 741,682 per case. After subtracting implementation costs, the corresponding net financial impacts (proxy-based) were JPY 156,969 per case for DESIGN-R 10–18 (implementation cost JPY 23,440) and JPY 676,480 per case for DESIGN-R ≥ 19 (implementation cost JPY 65,202). These findings suggest that severity-based prioritization may improve the practical value of electrical stimulation when resources are constrained.

Several limitations should be considered when interpreting these results. First, the ratio-based translation from 14-day wound-area dynamics to full time to heal necessarily relies on strong assumptions. In the base-case model, imputed baseline time to heal was divided by the healing acceleration ratio, which implicitly assumes that the proportional acceleration observed over 14 days remains constant throughout the entire healing trajectory. This assumption is unlikely to hold exactly in clinical practice, particularly for deep pressure injuries in which healing may be affected by infection, exudate, undermining, necrosis, and the local mechanical environment (pressure and shear) [1,4,24]. We therefore interpreted the base-case analysis as a transparent modeling approximation and supplemented it with conservative scenario analyses, including diminishing-acceleration and alternative ratio-summary approaches. Second, the healing acceleration ratio showed substantial uncertainty (bootstrap 95% UI 1.53–13.67), reflecting the instability that can arise when ratio-based measures are constructed from a small denominator in a small sample. Rather than treating *r* as the primary clinical estimate, we therefore positioned the crossover-appropriate within-subject difference (Δ) as the main short-term effectiveness result and used *r* only as a pragmatic translation parameter for the economic model. Additional median-based and trimmed-ratio sensitivity analyses were undertaken to examine whether extreme ratio values materially altered the interpretation. Third, baseline time to heal under placebo was assigned from published severity-stratified median healing times rather than directly observed in the trial cohort. Although this was a reasonable and transparent approach for linking short-term trial data to a longer-term decision model, medians from external cohorts do not function as directly observed means in the present sample. Accordingly, we examined the dependence of the findings on this assumption by varying imputed baseline healing time by ±20% and ±30%. The results indicate that the absolute size of projected days saved and monetary consequences is sensitive to the baseline healing-time input and should, therefore, be interpreted as scenario-based estimates rather than precise forecasts. Fourth, this study did not include a direct cost comparison between electrical stimulation and other existing treatment approaches used in routine pressure injury care. Accordingly, the present analysis should be interpreted as a within-model cost–consequence analysis of electrical stimulation under stated assumptions rather than as a comparative economic evaluation against alternative treatment strategies. In addition, several implementation cost inputs were parameterized using pragmatic assumptions rather than empirically derived real-world delivery data. These included the assumed device sharing pattern, consumable use per case, and additional staff time per treatment day. Although these assumptions were made transparently and examined in sensitivity analyses, they may not fully reflect variation in routine clinical practice. Future research should address these limitations through prospective studies that follow patients until complete healing and use time to heal as a primary endpoint. In parallel, economic data collection should move beyond a per-day approximation by measuring resource use directly (staff time, consumables, support surfaces, nutrition, complication-related care, and potential effects on length of stay) and applying setting-specific unit costs [8,9,12,21]. Given the severity-dependent patterns suggested here, future trials and observational studies should prespecify stratification by severity indicators (e.g., DESIGN-R category, depth, undermining, and infection status) and include multicenter data to improve external validity. Future studies should also compare electrical stimulation with existing treatment approaches used in routine pressure injury management, using a clearly defined comparative economic framework. Such analyses would clarify whether the modeled economic consequences of electrical stimulation differ relative to currently used care strategies. In addition, future implementation studies should obtain real-world application parameters from clinicians and facilities actually delivering treatment, including device utilization, consumable use, session frequency, and staff time requirements. Incorporating these empirically derived inputs would improve the realism and external validity of economic projections. Moreover, future economic analyses should integrate implementation costs and clinical uncertainty within the same resampling framework (e.g., recomputing net financial impact within each bootstrap draw) and explore alternative delivery scenarios (e.g., electrode replacement frequency, session frequency, wage rates, and device utilization across patient volumes) to better inform real-world adoption decisions.

Overall, this study illustrated the potential economic value of electrical stimulation therapy for deep PIs by translating healing acceleration into estimated reductions in time to heal and associated healing-related cost offsets (modeled gross offsets). By additionally accounting for device, consumables, and delivery time, the analysis suggests that electrical stimulation may be cost-offsetting under the base-case modeled assumptions. Notably, the break-even average implementation cost per treatment day was JPY 10,080, substantially higher than the estimated average implementation cost (~JPY 949/day), providing a pragmatic margin for real-world implementation under the model assumptions. The base-case device allocation (12 cases per device) should be viewed as a conservative, trial-anchored assumption. In routine practice, a device is typically used across a larger number of patients over its useful life. Accordingly, per-case device costs may be lower than in the base case when patient throughput is moderate to high. Beyond demonstrating potential clinical benefit, these findings may help inform pragmatic decisions regarding which patients to prioritize for electrical stimulation under limited resources.

## 5. Conclusions

This model-based analysis suggests that electrical stimulation (ES) may be associated with shorter projected pressure-injury healing times and a potentially cost-offsetting modeled net financial consequence at the facility level under stated assumptions, even after accounting for implementation costs (device, consumables, and additional staff time).

However, these projections depend materially on how short-term healing dynamics are translated into full time to heal and on externally sourced baseline healing-time assumptions. Prospective studies directly measuring time to heal, resource use, and costs are therefore needed to confirm the real-world economic implications.

## Figures and Tables

**Table 1:** Point estimates and 95% uncertainty intervals for clinical effectiveness inputs, healing-time translation outputs, and modeled economic outcomes.

Outcome	Point Estimate	95% Uncertainty Interval (Lower)	95% Uncertainty Interval (Upper)
Clinical effectiveness inputs			
Within-subject difference in daily wound area reduction, Δ (cm^2^/day) *	0.128	0.041	0.216
Healing acceleration ratio	3.52	1.53	13.67
Healing-time translation outputs			
Imputed baseline time to heal (days)	177.3	128.3	226.3
Estimated time to heal under stimulation (days)	50.4	12.4	113.8
Days saved (days)	126.9	63.5	164.9
Modeled economic outcomes			
Gross monetary value offset (proxy-based) (JPY/case; JPY 4000/day monetary value proxy)	507,723	253,854	659,434
Electrical stimulation implementation cost (JPY/case)	47,794	19,348	95,394
Average implementation cost (JPY/day)	949	836	1522
Net financial impact (proxy-based) (JPY/case)	459,929	158,459	640,086

* Abbreviations: ES, electrical stimulation; PI, pressure injury. Δ denotes the crossover-appropriate primary clinical effect, defined as the within-subject difference in daily wound area reduction between periods (ES minus placebo). The interval for Δ is a two-sided 95% confidence interval from paired analysis. The healing acceleration ratio (*r*) was treated as a secondary translation parameter for economic modeling and was calculated as the ratio of mean daily wound area reduction during the ES period to that during the placebo period. T_placebo represents imputed baseline time to heal under placebo/usual care, assigned using published median healing times stratified by DESIGN-R score and PI depth. T_ES was calculated as T_ES = T_placebo/*r* and days saved as T_placebo − T_ES. Gross offset was calculated as days saved × JPY 4000/day (base-case per-day healing-related monetary value proxy). ES implementation cost included a fixed per-case component (device allocation across 12 cases plus patient-specific consumables; JPY 10,017/case) and a variable labor component (JPY 750/day) applied over T_ES days. Net financial impact was calculated as gross offset − implementation cost. All interval estimates for *r* and model outcomes are 95% uncertainty intervals obtained by patient-level nonparametric bootstrap resampling (10,000 iterations), recomputing downstream quantities within each iteration. Monetary values should be interpreted as modeled facility-level financial proxies rather than directly observed economic costs or savings.

**Table 2:** Severity-stratified modeled healing time and economic outcomes for deep pressure injuries.

DESIGN-R Stratum (Deep)	*n*	T_ES, Days	Days Saved, Days	Gross Monetary Value Offset, JPY/Case	ES Implementation Cost, JPY/Case	Net Financial Impact, JPY/Case
10–18	5	17.9 (4.4–40.4)	45.1 (22.6–58.6)	180,409 (90,202–234,317)	23,440 (13,333–40,354)	156,969 (49,848–220,984)
≥19	7	73.6 (18.2–166.3)	185.4 (92.7–240.8)	741,682 (370,829–963,302)	65,202 (23,648–134,736)	676,480 (236,093–939,654)

Uncertainty intervals (UIs) were obtained by patient-level nonparametric bootstrap resampling (10,000 iterations), recomputing *r* and all downstream outcomes within each iteration. Baseline T_placebo was fixed to the stratum-specific published median time to heal. (Sanada et al. [15]).

## Data Availability

The data that support the findings of this study are available on reasonable request from the corresponding author. The data are not publicly available due to privacy concerns and ethical restrictions associated with the secondary use of clinical patient data.

## Supplementary Materials

The following supporting information can be downloaded at: https://www.mdpi.com/article/10.3390/healthcare14101269/s1, Table S1: Exploratory crossover diagnostic estimates for treatment, period, and sequence effects; Table S2: Sensitivity analyses using alternative ratio-based translation parameters for economic modeling; Table S3: One-way sensitivity analysis for the per-day healing-related monetary value proxy.
