# Supplementary material for: Model-Based Financial Consequences of Electrical Stimulation Therapy for Pressure Injury Healing
Source: Healthcare (Basel). 2026 May 7;14(10):1269. doi: 10.3390/healthcare14101269 (PMC13205805; doi:10.3390/healthcare14101269)
Supplement: Supplementary file 1 [file healthcare-14-01269-s001.zip › healthcare-4140991-supplementary.pdf]

Table S1: Exploratory crossover diagnostic estimates for treatment, period, and sequence effects.

Parameter	Estimate	95% confidence interval lower	95% confidence interval upper	p value
Treatment effect (ES vs placebo)	0.127	0.052	0.203	0.002
Period effect (period 2 vs period 1)	-0.045	-0.120	0.031	0.234
Sequence effect (placebo→ES vs ES→placebo)	-0.005	-0.081	0.070	0.883

Exploratory crossover diagnostic estimates were obtained using a simple 2 × 2 crossover linear model including treatment, period, and sequence terms. The outcome was daily wound area reduction (cm^2/day). The treatment effect is expressed as ES vs placebo. Because the study included only 12 patients, these diagnostic estimates should be interpreted cautiously and were provided primarily for transparency rather than for definitive inference.

Table S2: Sensitivity analyses using alternative ratio-based translation parameters for economic modeling.

**Scenario**	**Healing acceleration ratio, r**	**Estimated time-to-heal under ES, T_ES (days)**	**Days saved (days)**	**Gross offset (JPY/case)**	**Net financial impact (JPY/case)**
Base-case mean-based ratio	3.52	50.4	126.9	507,723	459,929
Median-based ratio	6.00	29.6	147.8	591,000	558,821
Trimmed-ratio scenario*	4.24	41.8	135.5	542,093	500,744

Abbreviations: ES, electrical stimulation.

The base-case mean-based ratio was calculated as the mean daily wound area reduction during the ES period divided by that during the placebo period. The median-based ratio was calculated as the median daily wound area reduction during the ES period divided by that during the placebo period. The trimmed-ratio scenario was based on the mean of bootstrap-derived ratio values after excluding extreme values below the 2.5th percentile and above the 97.5th percentile of the bootstrap distribution.

For each scenario, estimated time-to-heal under ES was calculated as T_ES = T_placebo / r, using the mean imputed baseline time-to-heal under placebo/usual care (T_placebo = 177.3 days). Days saved were calculated as T_placebo − T_ES. Gross offset was calculated as days saved × JPY 4,000/day. Net financial impact was calculated as gross offset − implementation cost, where implementation cost = JPY 10,017 + (JPY 750/day × T_ES). These analyses were conducted to assess the sensitivity of modeled outcomes to alternative summaries of the ratio-based translation parameter.

Table S3: One-way sensitivity analysis for the per-day healing-related monetary value proxy.

Per-day healing-related monetary value proxy (JPY/day)	Gross offset (JPY/case)	Net financial impact (JPY/case)
3,000	380,792	332,998
4,000 (base case)	507,723	459,929
5,000	634,654	586,860
7,000	888,515	840,721
8,000	1,015,446	967,652
9,000	1,142,377	1,094,583
10,000	1,269,308	1,221,514

Gross offset and net financial impact were recalculated by varying the per-day healing-related monetary value proxy while holding the base-case healing-time translation and ES implementation cost structure constant. The base-case proxy value was JPY 4,000/day.

This analysis was intended to examine the extent to which modeled monetary consequences depended on the assumed daily proxy for healing-related resource use. Monetary values should be interpreted as scenario-based modeled outputs rather than directly observed costs or savings.
